# Supplementary material for: Global computational alignment of tumor and cell line transcriptional profiles
Source: Nat Commun. 2021 Jan 4;12:22. doi: 10.1038/s41467-020-20294-x (PMC7782593; doi:10.1038/s41467-020-20294-x)
Supplement: Supplementary file 1 — Supplementary Information [file 41467_2020_20294_MOESM1_ESM.pdf]

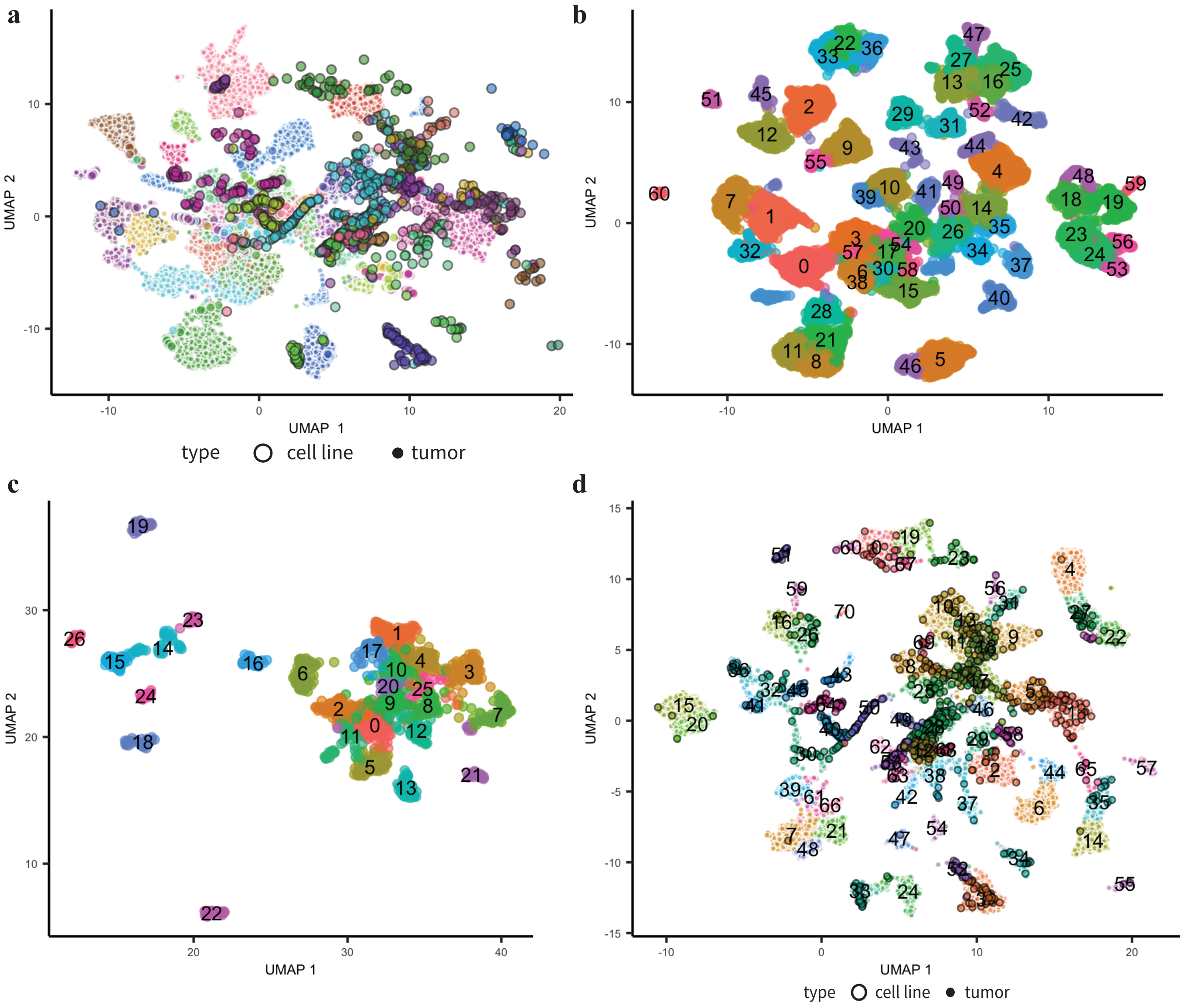

Supplementary Figure 1. 2D Projections of cell lines and tumor samples. (n = 1,249 cell lines, n = 12,236 tumors) **a** Projection of cell lines and tumors after COMBAT correction colored by cancer lineage. Cancers of the same type do not align between cell lines and tumors. **b** Clustering of the uncorrected tumor expression data colored and labeled by the clusters identified. **c** Clustering of the uncorrected cell line expression data colored and labeled by the clusters identified. **d** Clustering of the Celligner-aligned tumor and cell line expression data colored and labeled by the clusters identified.

a

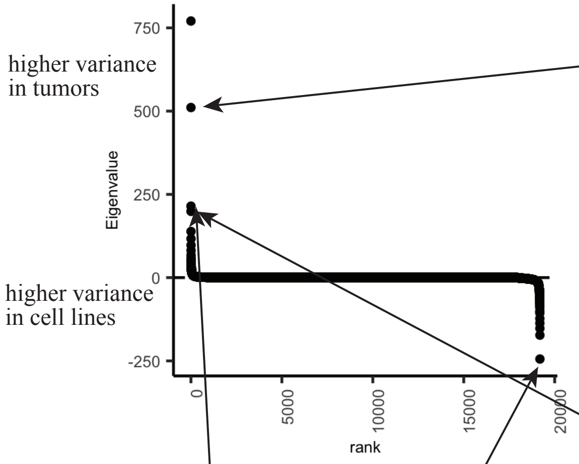

b

pathway	adjusted pval	NES
GO_ADAPTIVE_IMMUNE_RESPONSE	0.000469	3.26
GO_HUMORAL_IMMUNE_RESPONSE	0.000469	3.15
GO_LEUKOCYTE_MEDIATED_IMMUNITY	0.000469	3.09
GO_ADAPTIVE_IMMUNE_RESPONSE_BASED	0.000469	3.07
GO_LYMPHOCYTE_MEDIATED_IMMUNITY	0.000469	3.07
GO_RNA_SPLICING_VIA_TRANSESTERIFICATION	0.000636	-2.30
GO_SISTER_CHROMATID_SEGREGATION	0.000587	-2.30
GO_PEPTIDYL_LYSINE_MODIFICATION	0.000647	-2.26
GO_RIBONUCLEOPROTEIN_COMPLEX_LOCALIZATION	0.000556	-2.25
GO_RNA_LOCALIZATION	0.000592	-2.24

c

pathway	adjusted pval	NES
GO_ESTABLISHMENT_OF_PROTEIN_LOCALIZATION	0.00129	1.93
GO_MITOCHONDRIAL_RESPIRATORY_CHAIN_COMPLEX	0.00129	1.90
GO_PROTEIN_LOCALIZATION_TO_ENDOPLASMIC	0.00129	1.88
GO_NUCLEAR_TRANSCRIBED_MRNA_CATABOLIC	0.00129	1.88
GO_OXIDATIVE_PHOSPHORYLATION	0.00129	1.85
GO_MAGNESIUM_ION_TRANSMEMBRANE_TRANSPORT	0.02460	-2.25
GO_ESTABLISHMENT_OF_PROTEIN_LOCALIZATION	0.02310	-2.19
GO_MAGNESIUM_ION_TRANSPORT	0.05230	-2.18
GO_PHAGOCYTOSIS_RECOGNITION	0.02780	-2.10
GO_CHROMATIN_SILENCING_AT_RDNA	0.04250	-1.97

d

pathway	adjusted pval	NES
GO_EXTRACELLULAR_STRUCTURE_ORGANIZATION	0.000177	2.87
GO_MUSCLE_CONTRACTION	0.000177	2.76
GO_MUSCLE_SYSTEM_PROCESS	0.000177	2.72
GO_REGULATION_OF_OSSIFICATION	0.000177	2.67
GO_MUSCLE_ORGAN_DEVELOPMENT	0.000177	2.64
GO_SISTER_CHROMATID_SEGREGATION	0.000177	-3.01
GO_NUCLEAR_CHROMOSOME_SEGREGATION	0.000177	-2.94
GO_CHROMOSOME_SEGREGATION	0.000177	-2.94
GO_SISTER_CHROMATID_COHESION	0.000177	-2.83
GO_MITOTIC_SISTER_CHROMATID_SEGREGATION	0.000177	-2.73

e

pathway	adjusted pval	NES
GO_RESPONSE_TO_TYPE_I	0.000541	2.80
GO_DEFENSE_RESPONSE_TO_VIRUS	0.000541	2.50
GO_NEGATIVE_REGULATION_OF_VIRAL	0.000541	2.45
GO_NEGATIVE_REGULATION_OF_VIRAL	0.000541	2.43
GO_INTERFERON_GAMMA_MEDIATED_SIGNALING	0.000541	2.42
GO_DNA_DEPENDENT_DNA_REPLICATION	0.000924	-2.59
GO_RNA_SPLICING_VIA_TRANSESTERIFICATION	0.001150	-2.53
GO_DNA_REPLICATION	0.001040	-2.52
GO_DNA_RECOMBINATION	0.001040	-2.49
GO_RIBONUCLEOPROTEIN_COMPLEX_LOCALIZATION	0.000934	-2.47

Supplementary Figure 2. contrastive Principle Component Analysis (cPCA) a cPCA eigenvalue spectrum. **b** GSEA of the second, **c** third, **d** and fourth cPCs, which are higher variance in tumors. **e** GSEA of the top cell line specific cPC. P-values are based on a gene-permutation test and adjusted using the Benjamini-Hochberg procedure (see Methods, 'Gene set enrichment analysis').

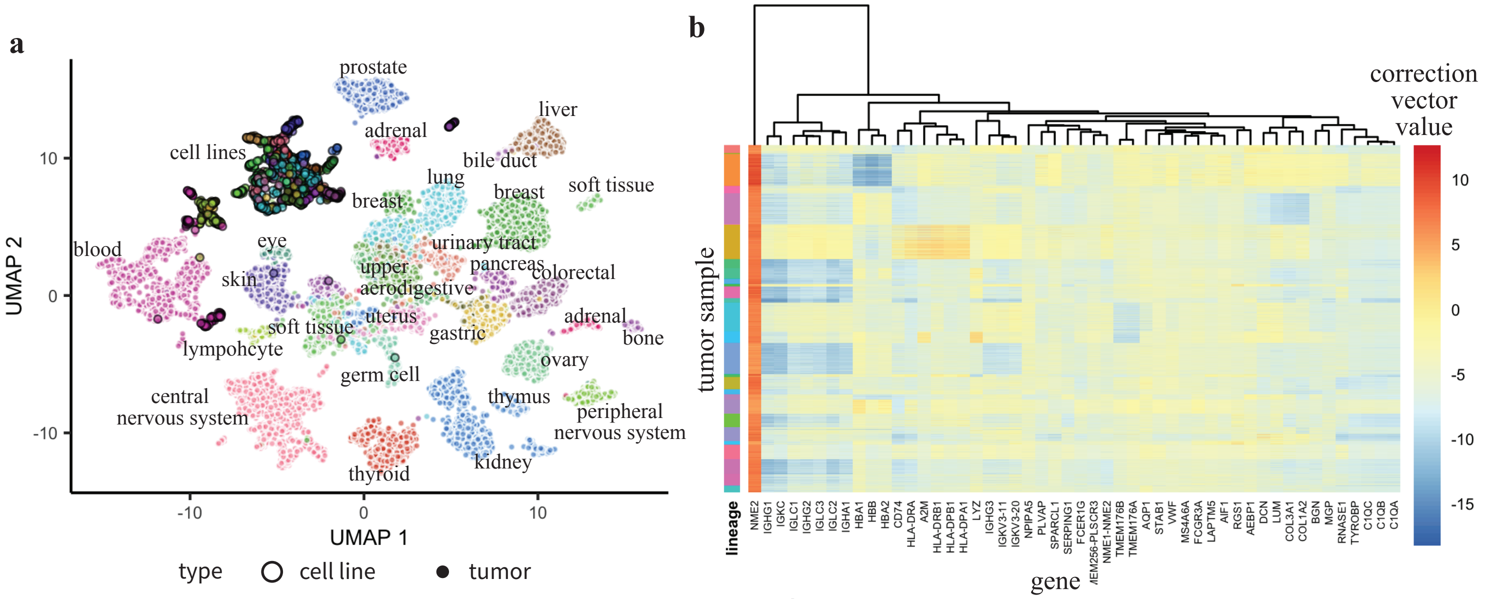

c

GSEA of average MNN correction vectors

pathway	adjusted pval	NES	higher expression in
GO_HUMORAL_IMMUNE_RESPONSE	0.000408	-2.83	tumors
GO_PROTEIN_ACTIVATION_CASCADE	0.000408	-2.78	tumors
GO_COMPLEMENT_ACTIVATION	0.000408	-2.75	tumors
GO_HUMORAL_IMMUNE_RESPONSE_MEDIATED	0.000408	-2.74	tumors
GO_B_CELL_MEDIATED_IMMUNITY	0.000408	-2.70	tumors
GO_SISTER_CHROMATID_SEGREGATION	0.000574	3.12	cell lines
GO_CHROMOSOME_SEGREGATION	0.000635	3.08	cell lines
GO_NUCLEAR_CHROMOSOME_SEGREGATION	0.000597	3.08	cell lines
GO_SISTER_CHROMATID_COHESION	0.000547	2.91	cell lines
GO_MITOTIC_SISTER_CHROMATID_SEGREGATION	0.000537	2.90	cell lines

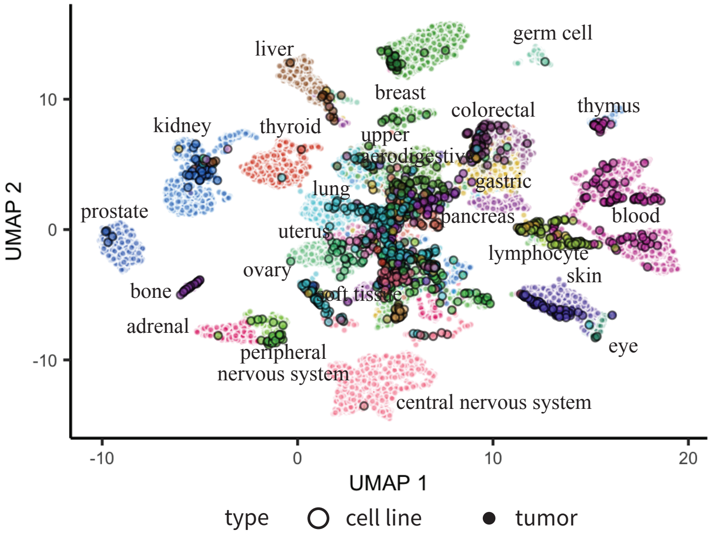

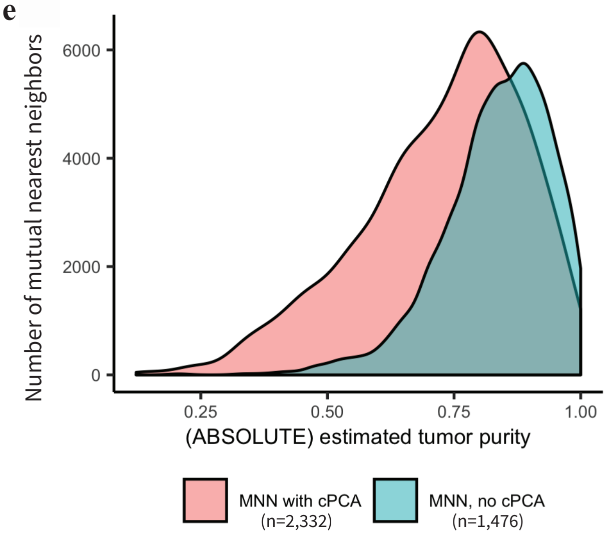

Supplementary Figure 3. cPCA and MNN correction. **a** Projection of cell lines (n=1,249) and tumors (n=12,236) using just cPCA correction colored by cancer lineage. **b** Heatmap of the MNN correction vectors per tumor sample, showing the 50 genes with the highest absolute average correction vector values. **c** GSEA of the average MNN correction vectors. P-values are based on a gene-permutation test and adjusted using the Benjami-ni-Hochberg procedure (see Methods, ‘Gene set enrichment analysis’). **d** Projection of cell line (n=1,249) and tumor (n=12,236) data using just MNN correction colored by cancer type. **e** Running cPCA before MNN increases the number of identified mutual nearest neighbors, especially for lower purity tumors.

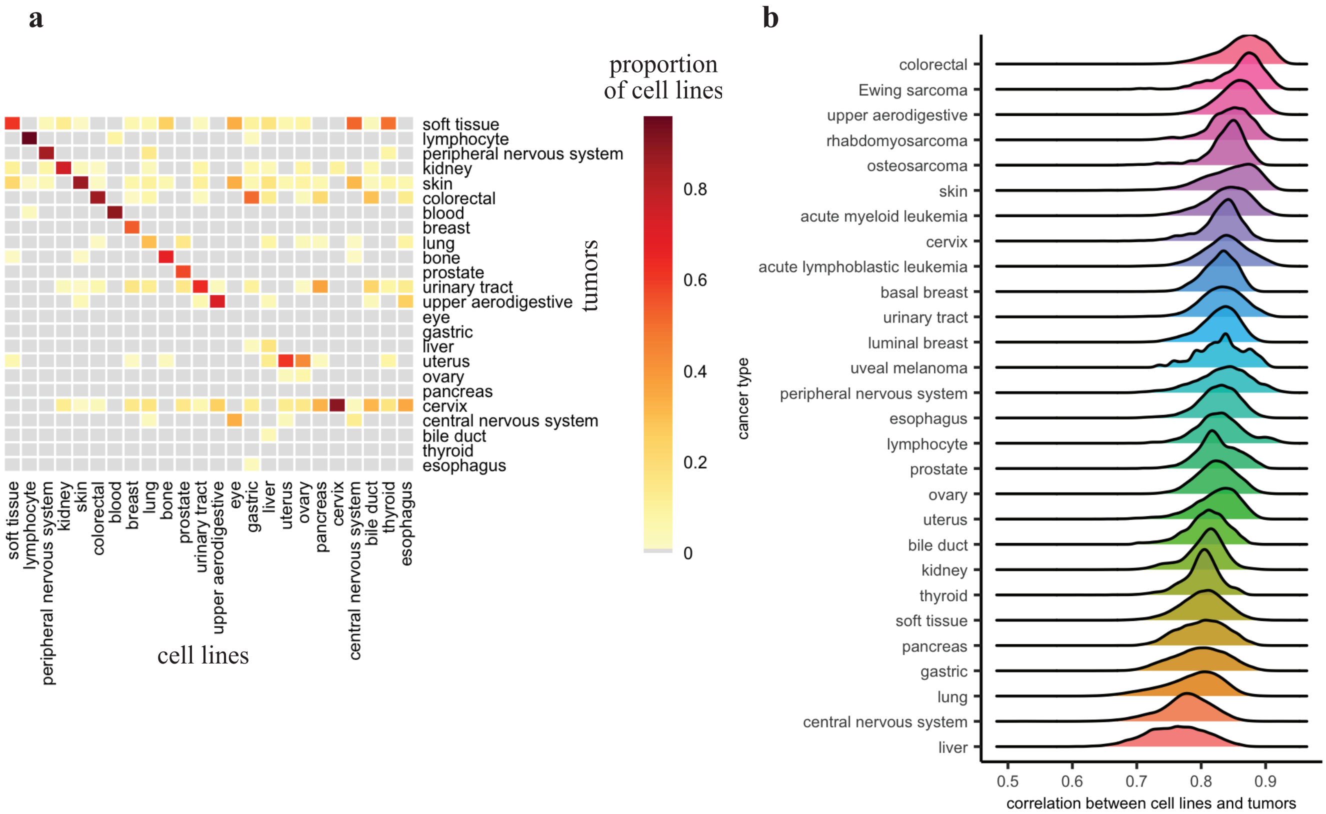

Supplementary Figure 4. Uncorrected classification of cell lines by tumor type a Proportion of cell lines (n=1,150) that are classified as each tumor type using uncorrected data. 49% of cell lines with corresponding types in the tumor data matched to tumors of the same type. **b** Distribution of correlations between cell lines (n=1,135) and tumors (n=11,413) of the same (sub)type using uncorrected data.

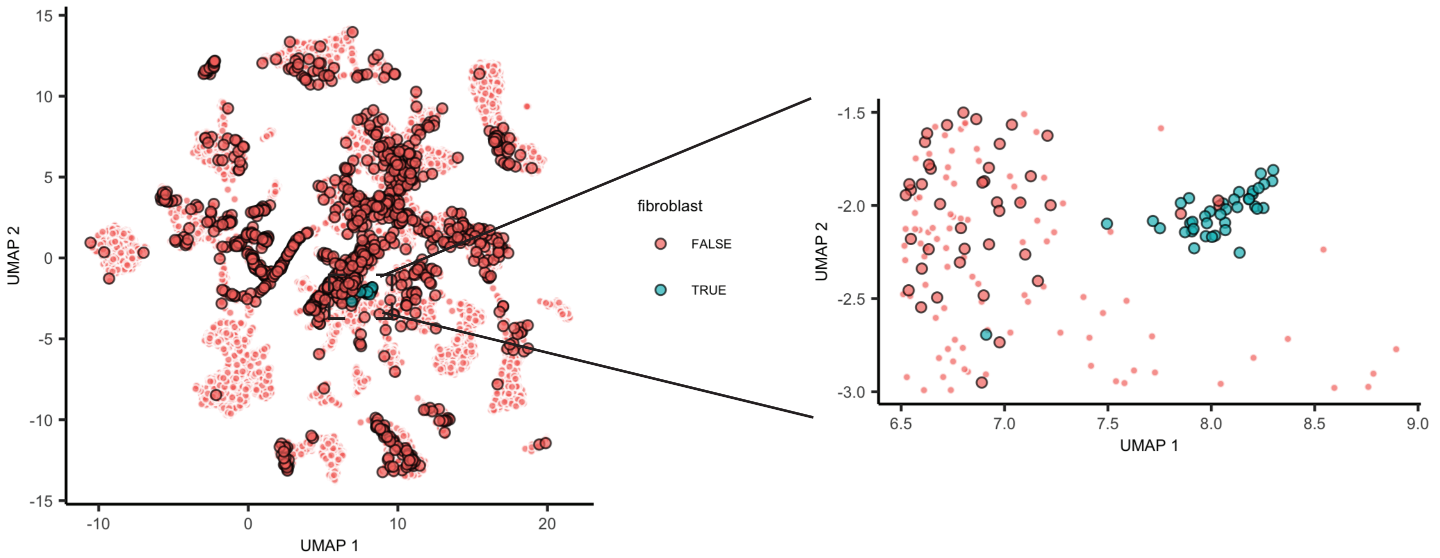

Supplementary Figure 5. Fibroblast cell lines. 38/39 of the fibroblast cell lines clustered together, with only 5 samples (4 cell lines and 1 tumor sample) not annotated as fibroblasts.

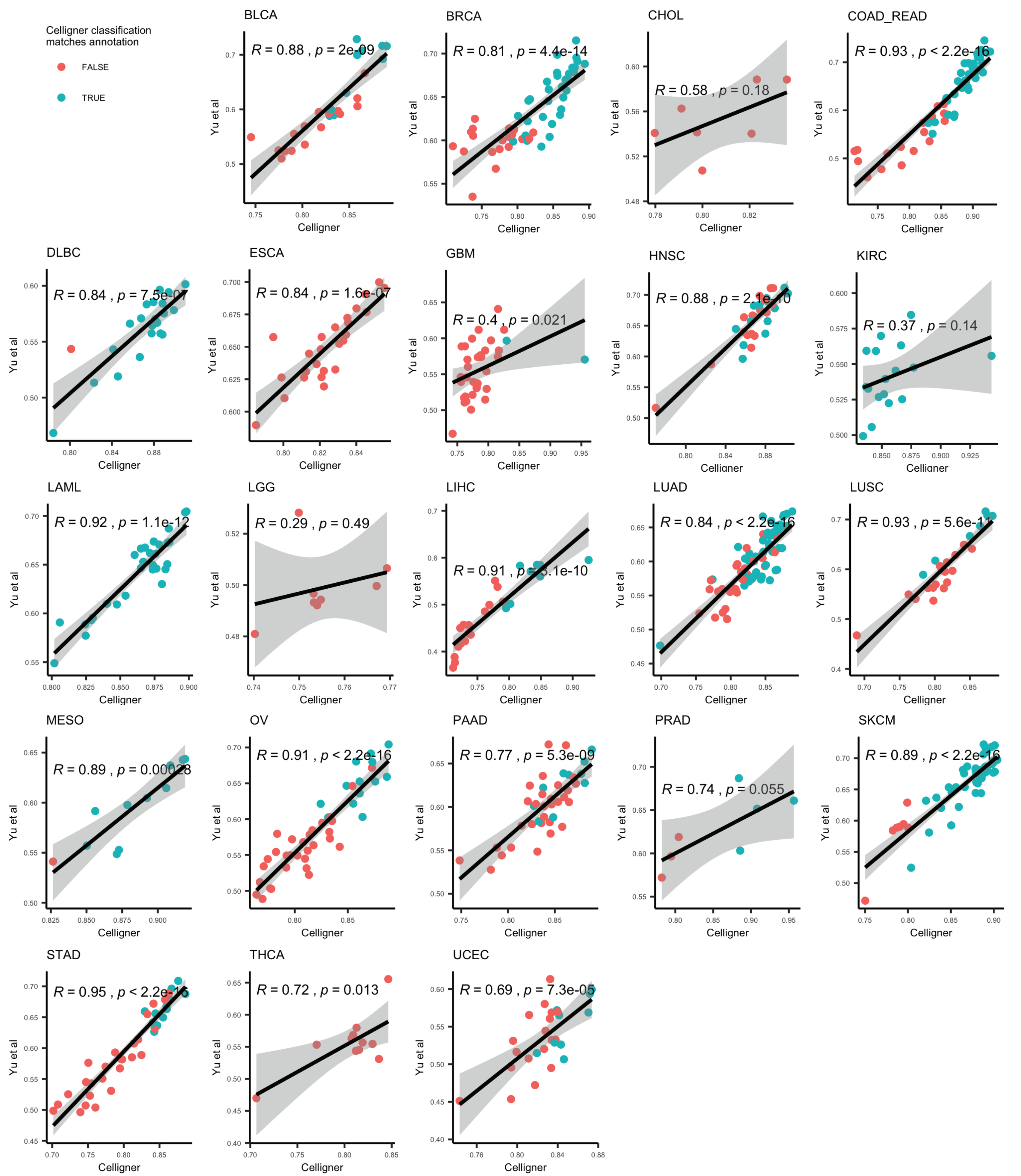

Supplementary Figure 6. Comparison of results from Celligner and Yu et al. Each plot shows on the x-axis the median correlation in Celligner-aligned data between cell lines annotated as that type to tumors of that type and on the y-axis the median correlation between cell lines annotated as that type to tumors of that type, as calculated by Yu et al., 2019. Samples are colored by whether or not they were classified by Celligner nearest neighbors classifications as the annotated type. The black line shows the linear regression trend line, with the 95% confidence interval shown in gray and the Pearson correlation and associated p-value shown for each plot (n = 666 cell lines, n = 7,656 tumors).

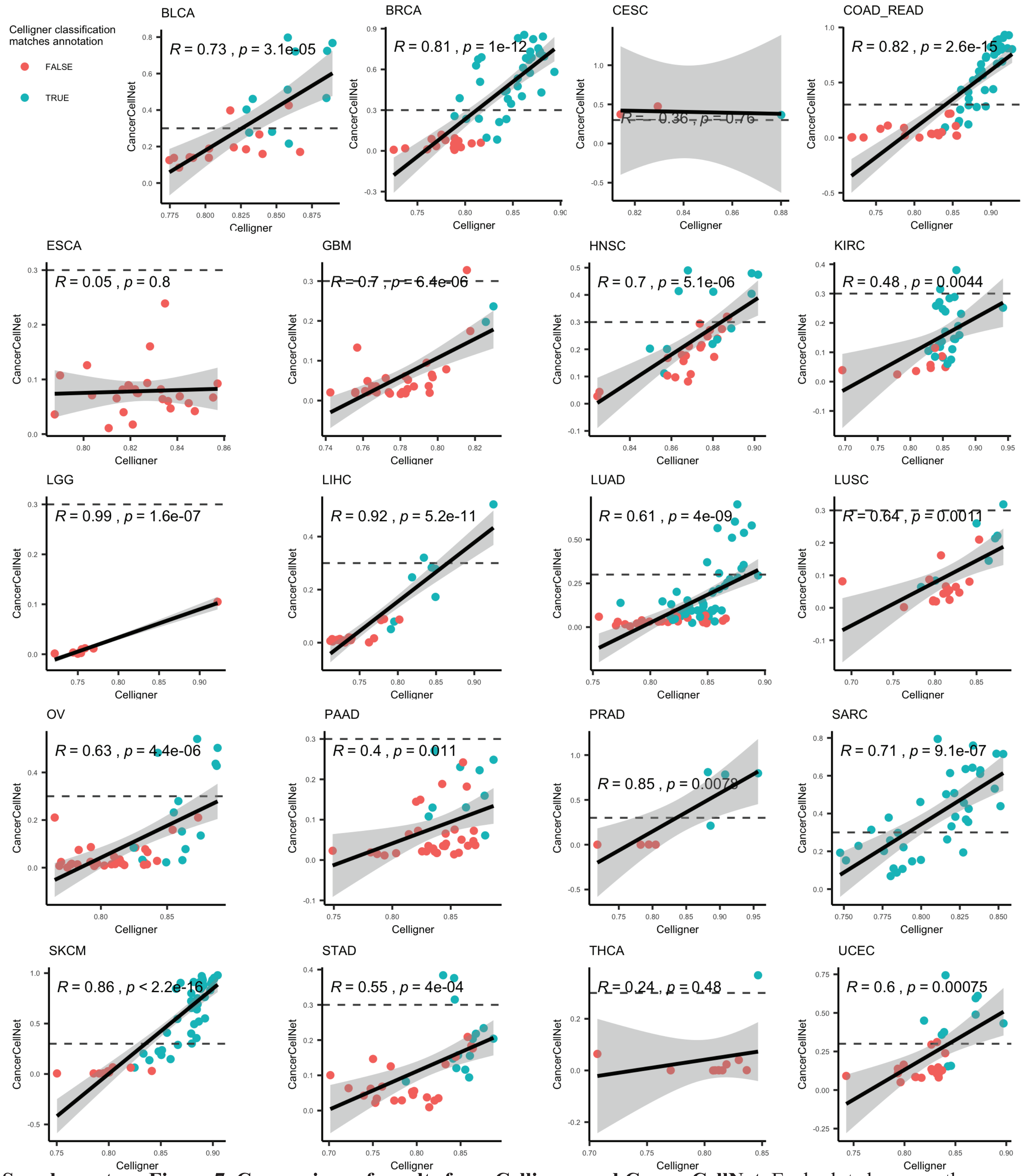

Supplementary Figure 7. Comparison of results from Celligner and CancerCellNet. Each plot shows on the x-axis the median correlation in Celligner-aligned data between cell lines annotated as that type to tumors of that type and on the y-axis the probability from CancerCellNet of cell lines being classified as that type. Samples are colored by whether they were classified by Celligner nearest neighbors classifications as the annotated type. The dashed line is added at 0.3 to mark the classification threshold from CancerCellNet and the black solid line shows the linear regression trend line, with the 95% confidence interval shown in gray and the Pearson correlation and associated p-value shown for each plot (n = 657 cell lines, n = 8,825 tumors).

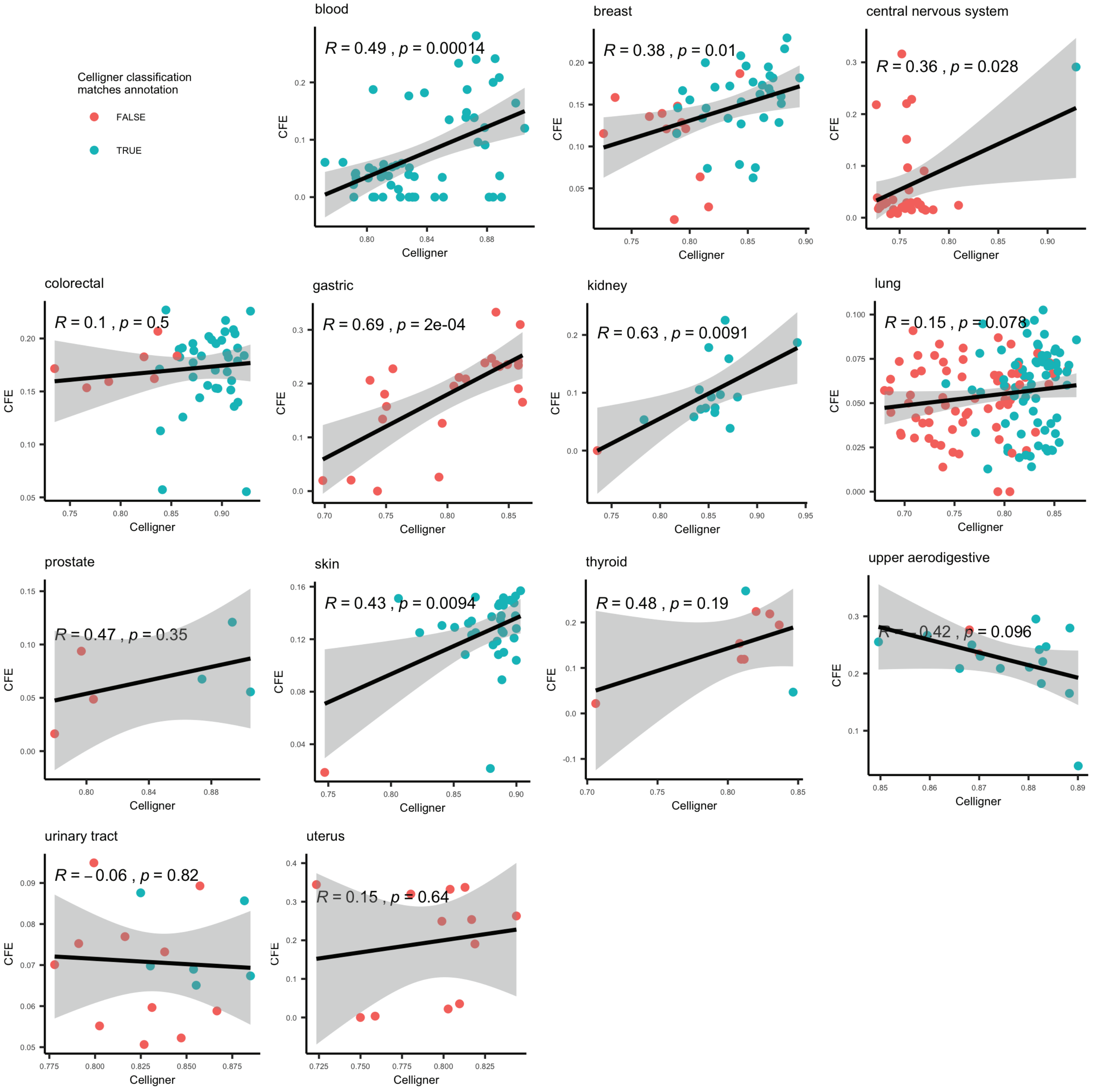

Supplementary Figure 8. Comparison of results from Celligner and Cancer Function Events (CFEs). Each plot shows on the x-axis the median correlation in Celligner-aligned data between cell lines annotated as that type to tumors of that type and on the y-axis the median Jaccard similarity between cell lines annotated as that type to tumors of that type, calculated using the CFEs defined by Iorio et al., 2016. Plots only include samples included both in the CFE data and Celligner data (n=459 cell lines, n=2448 tumors). Samples are colored by whether they were classified by Celligner nearest neighbors classifications as the annotated type. The black line shows the linear regression trend line, with the 95% confidence interval shown in gray and the Pearson correlation and associated p-value shown for

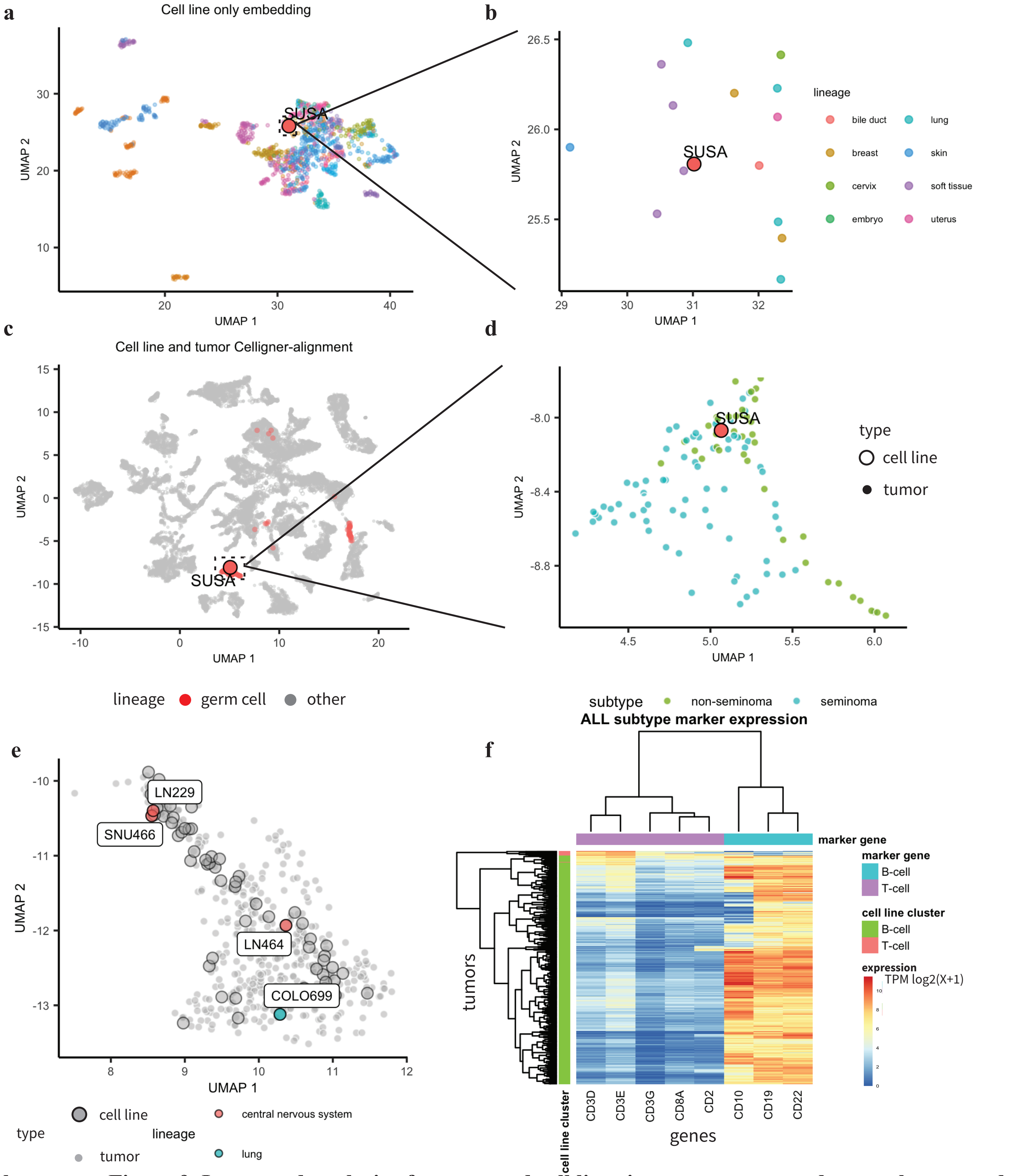

Supplementary Figure 9. Integrated analysis of tumors and cell lines increases power to detect subtypes and identify misannotated samples. We computed a 2D UMAP embedding of just the cell line (n = 1,249) RNA-Seq data and the Celligner-aligned data (n = 1,249 cell lines, n=12,236 tumors). **a, b** In the cell line only embedding, SUSA, the only testicular cell line in the data, clusters near cell lines of a variety of tissue types, **c** while in the Celligner-aligned embedding SUSA clusters with the germ cell tumors, **d** and its nearest tumors neighbors are primarily non-seminoma testicular cancer samples. **e** Four cell lines which are not annotated as skin cell lines, but cluster with the melanoma cluster (n = 59 cell lines, n = 442 tumors). **f** Clustering of ALL tumor samples (n = 525 tumors) by expression of ALL subtype marker genes agrees well with the clustering of those samples with ALL T-cell and B-cell cell lines.

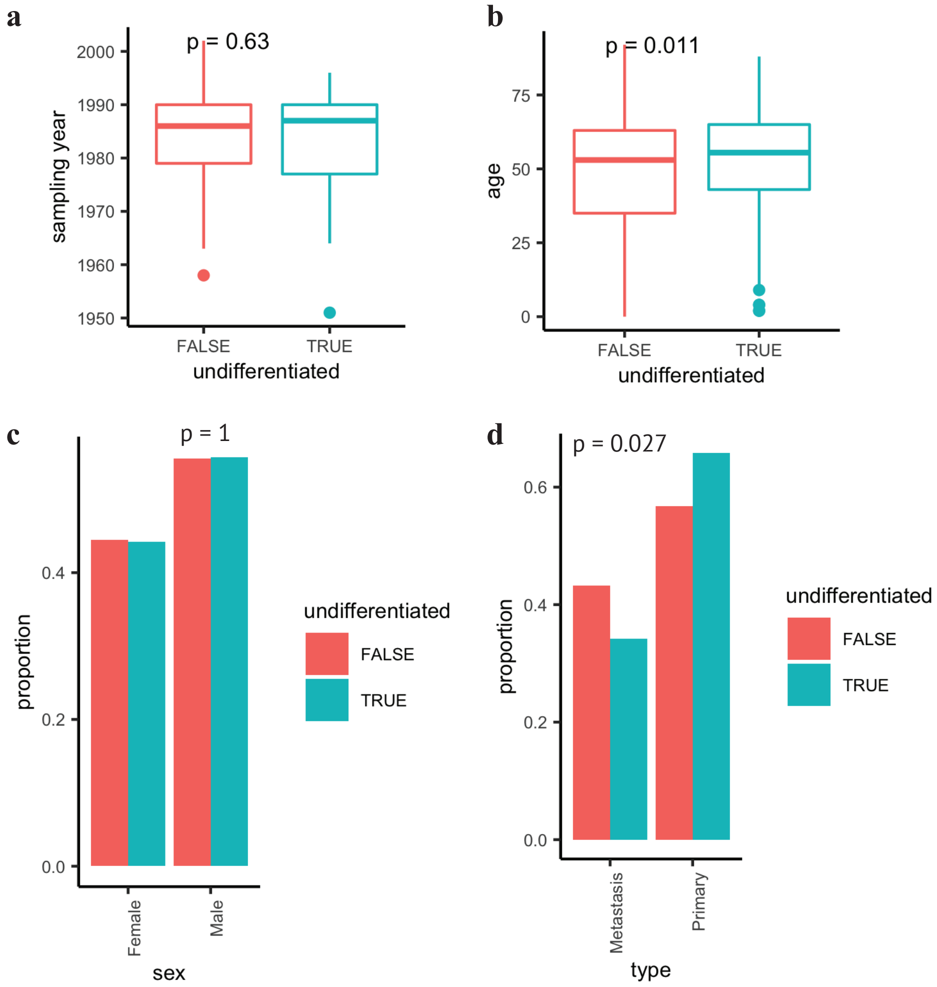

Supplementary Figure 10. Features of the undifferentiated cell lines. a The age of the cell lines (year that the cell line was derived) is not significantly different (two-sided wilcoxon test, p=0.63) between the undifferentiated cell lines (n = 69 cell lines) and the remaining cell lines (n = 273 cell lines). **b** The age of the patient from which the cell lines were derived was significantly different (two-sided wilcoxon test, p=0.011) between cell lines that fell in the undifferentiated cluster (n = 168 cell lines) compared to cell lines that did not (n = 716 cell lines), although the difference in means was quite small (5.3 years). Boxplots are shown with the box representing the median (center line), 25th (lower line), and 75th (upper line) percentiles and whiskers showing 1.5x interquartile range. **c** The proportion of male and female cell lines is not significantly different (two proportion z-test, p=1) between cell lines in the undifferentiated cluster (n = 100 female cell lines, n = 126 male cell lines) and cell lines not in the undifferentiated cluster (n = 406 female cell lines, n = 507 male cell lines). **d** The proportion of cell lines derived from primary patient samples was signficantly higher (two proportion z-test, p=.027) for cell lines in the undifferentiated cluster (n = 129 primary cell lines, n = 67 metastatic cell lines) compared to cell lines not in the undifferentiated cluster (n = 412 primary cell lines, n = 314 metastatic cell lines), although this may be confounded by differences in lineage.

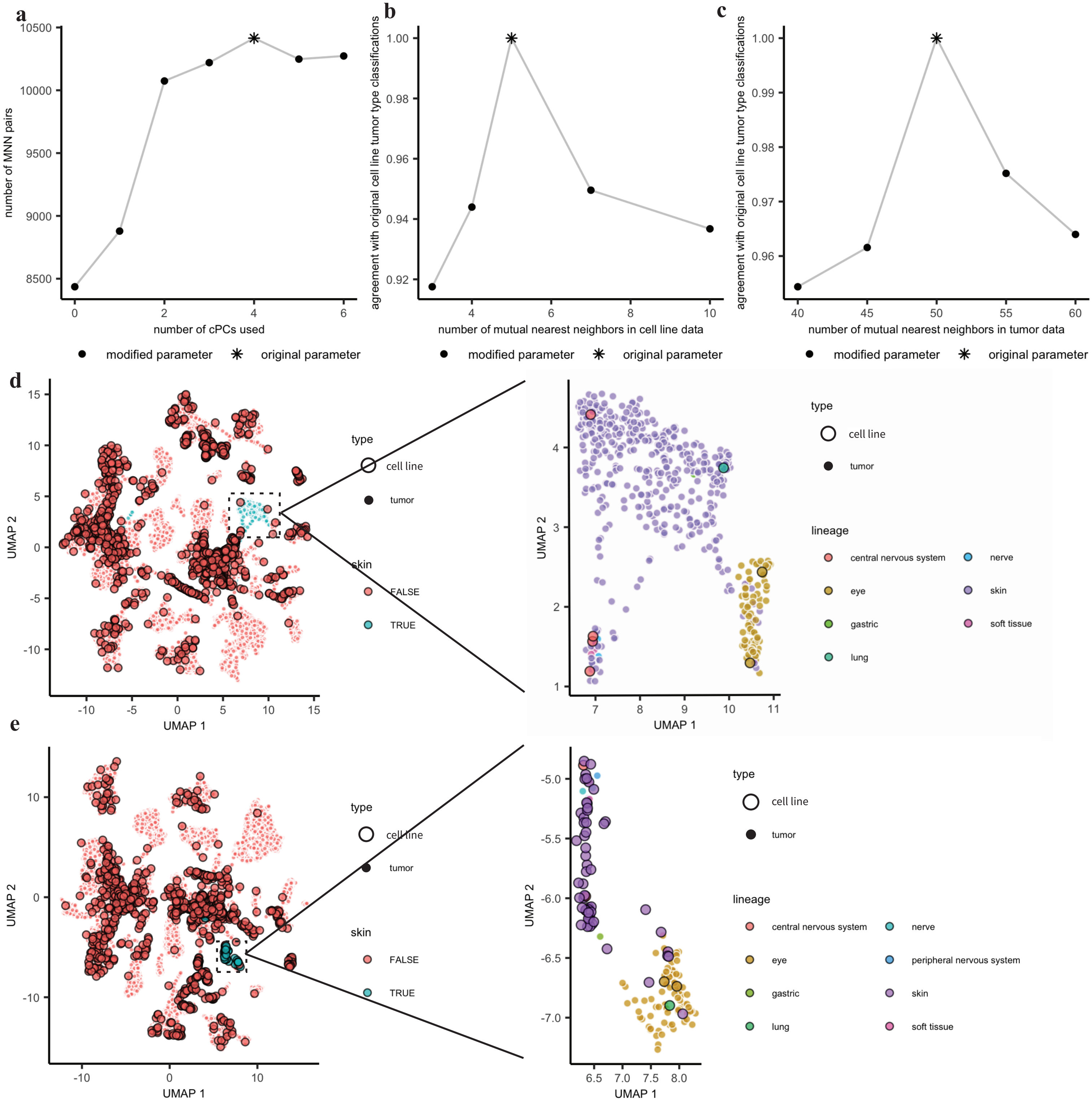

Supplementary Figure 11. Celligner parameter selection and robustness. a Regressing out the top four cPCs with higher variance in the tumor data increased the number of mutual nearest neighbors. **Celligner output is robust to alterations in parameters. b, c** In order to evaluate the robustness of the output we varied one parameter at a time, keeping all other parameters fixed, then compared the tumor type classifications for each cell line (see Methods) to the original classifications. The parameters that we tested were the k parameters used as input to MNN correction. **Celligner is robust to removal of data. d** We also tested the stability of the output to removal of a subset of the data. We removed all of the cell lines annotated as skin (n = 68) and re-ran the alignment. We saw that even without these cell lines the skin tumors (n = 476) formed a clear cluster, and cell lines that we believe are mis-annotated continued to cluster with the skin tumors. **e** We also tested removing the skin tumors (n = 476) and re-running the alignment. In this case, the skin cell lines (n = 68) clustered near the uveal melanoma samples, but primarily formed a separate cluster without tumors.

References

1. Johnson, W. E., Li, C. & Rabinovic, A. Adjusting batch effects in microarray expression data using empirical Bayes methods. Biostatistics 8, 118–127 (2007).
2. Yu, K. et al. Comprehensive transcriptomic analysis of cell lines as models of primary tumors across 22 tumor types. Nat. Commun. 10, 3574 (2019).
3. Peng, D. et al. Evaluating the transcriptional fidelity of cancer models. BioRxiv (2020). doi:10.1101/2020.03.27.012757
4. Iorio, F. et al. A landscape of pharmacogenomic interactions in cancer. Cell 166, 740–754 (2016).
