# Supplementary material for: Global computational alignment of tumor and cell line transcriptional profiles
Source: Nat Commun. 2021 Jan 4;12:22. doi: 10.1038/s41467-020-20294-x (PMC7782593; doi:10.1038/s41467-020-20294-x)
Supplement: Supplementary file 3 — Description of Additional Supplementary Files [file 41467_2020_20294_MOESM3_ESM.pdf]

Description of Additional Supplementary Files

File Name: Supplementary Data 1
Description: Provides a list of all samples (tumors and cell lines) used in this analysis, and relevant metadata (lineage, subtype, UMAP coordinates, etc.).

File Name: Supplementary Data 2
Description: List of differentially expressed genes used in mutual nearest neighbors batch correction.
