# Supplementary material for: Global computational alignment of tumor and cell line transcriptional profiles
Source: Nat Commun. 2021 Jan 4;12:22. doi: 10.1038/s41467-020-20294-x (PMC7782593; doi:10.1038/s41467-020-20294-x)
Supplement: Supplementary file 6 — Reporting Summary [file 41467_2020_20294_MOESM6_ESM.pdf]

nature research

Corresponding author(s): James McFarland

Last updated by author(s): Oct 16, 2020

Reporting Summary

Nature Research wishes to improve the reproducibility of the work that we publish. This form provides structure for consistency and transparency in reporting. For further information on Nature Research policies, see our Editorial Policies and the Editorial Policy Checklist.

Statistics

For all statistical analyses, confirm that the following items are present in the figure legend, table legend, main text, or Methods section.

n/a	Confirmed	
☐	☒	The exact sample size (*n*) for each experimental group/condition, given as a discrete number and unit of measurement
☐	☒	A statement on whether measurements were taken from distinct samples or whether the same sample was measured repeatedly
☐	☒	The statistical test(s) used AND whether they are one- or two-sided<br>*Only common tests should be described solely by name; describe more complex techniques in the Methods section.*
☐	☒	A description of all covariates tested
☐	☒	A description of any assumptions or corrections, such as tests of normality and adjustment for multiple comparisons
☐	☒	A full description of the statistical parameters including central tendency (e.g. means) or other basic estimates (e.g. regression coefficient) AND variation (e.g. standard deviation) or associated estimates of uncertainty (e.g. confidence intervals)
☐	☒	For null hypothesis testing, the test statistic (e.g. *F*, *t*, *r*) with confidence intervals, effect sizes, degrees of freedom and *P* value noted<br>*Give P values as exact values whenever suitable.*
☒	☐	For Bayesian analysis, information on the choice of priors and Markov chain Monte Carlo settings
☒	☐	For hierarchical and complex designs, identification of the appropriate level for tests and full reporting of outcomes
☐	☒	Estimates of effect sizes (e.g. Cohen's *d*, Pearson's *r*), indicating how they were calculated

Our web collection on statistics for biologists contains articles on many of the points above.

Software and code

Policy information about availability of computer code

Data collection	No software was used.
Data analysis	Adobe Illustrator version 24<br>Microsoft Word version 15.36<br>Microsoft Excel version 16.16.20<br>RStudio version 1.2.5033<br>R version 3.6.2 (2019-12-12)<br>R packages: here (v 0.1), tidyverse (v 1.3.0), reshape2 (v 1.4.3), plyr (v 1.8.5), data.table (v 1.12.8), Seurat (v 3.1.2), pheatmap (v 1.0.12), pdist (v 1.2), gridExtra (v 2.3), ggpubr (v 0.2.4), grDevices (v 3.6.2), RColorBrewer (v 1.1.2), FNN (v 1.1.3), ggrepel (v 0.8.1), ggridges (v 0.5.1), limma (v 3.42.0), edgeR (v 3.28.0), batchelor (v 1.2.4), BiocParallel (v 1.20.1), sva (v 3.34.0), GSEABase (v 1.48.0), piano (v 2.2.0), fgsea (v 1.12.0), scran (v 1.16.0)<br><br>Code to create all figures and run the method is available here: https://github.com/broadinstitute/Celligner_ms

For manuscripts utilizing custom algorithms or software that are central to the research but not yet described in published literature, software must be made available to editors and reviewers. We strongly encourage code deposition in a community repository (e.g. GitHub). See the Nature Research guidelines for submitting code & software for further information.

Data

Policy information about availability of data

All manuscripts must include a data availability statement. This statement should provide the following information, where applicable:

- Accession codes, unique identifiers, or web links for publicly available datasets
- A list of figures that have associated raw data
- A description of any restrictions on data availability

The cell line gene expression data, genome-wide CRISPR-Cas9 knockout (KO) data, and drug sensitivity data are publicly available at depmap.org (files DepMap Public 19Q4 CCLE_expression_full.csv, DepMap Public 19Q4 Achilles_gene_effect.csv, PRISM Repurposing 19Q4 secondary-screen-replicate-collapsed-logfold-change.csv, respectively). The tumor gene expression data is publicly available at xenabrowser.net (from the Treehouse Public Expression Dataset v10). Output from the Celligner method is available at Figshare: https://figshare.com/articles/Celligner_data/11965269.

Field-specific reporting

Please select the one below that is the best fit for your research. If you are not sure, read the appropriate sections before making your selection.

[x] Life sciences [ ] Behavioural & social sciences [ ] Ecological, evolutionary & environmental sciences

For a reference copy of the document with all sections, see nature.com/documents/nr-reporting-summary-flat.pdf

Life sciences study design

All studies must disclose on these points even when the disclosure is negative.

Sample size	For characterization of cancer cell lines and tumors, no sample size was predetermined as the goal is to characterize all possible human cancers. At this time, gene expression data for 1,249 human cancer cell lines were available, and CRISPR-Cas9 KO data were available for 684 of those cell lines and drug sensitivity data were available for 440 of those cell lines. At this time gene expression data for 12,236 tumor samples were available.
Data exclusions	All available data from DepMap Public 19Q4 and Treehouse Public Expression Dataset v10 were used, no data was excluded.
Replication	Not applicable to this study, as this study does not include any new experiments.
Randomization	Not applicable to this study, as this study does not include randomized experimental groups.
Blinding	Not applicable to this study, as this study does include any manual group allocation.

Reporting for specific materials, systems and methods

We require information from authors about some types of materials, experimental systems and methods used in many studies. Here, indicate whether each material, system or method listed is relevant to your study. If you are not sure if a list item applies to your research, read the appropriate section before selecting a response.

Materials & experimental systems

n/a	Involved in the study	
[x]	[ ]	Antibodies
[x]	[ ]	Eukaryotic cell lines
[x]	[ ]	Palaeontology and archaeology
[x]	[ ]	Animals and other organisms
[x]	[ ]	Human research participants
[x]	[ ]	Clinical data
[x]	[ ]	Dual use research of concern

Methods

n/a	Involved in the study	
[x]	[ ]	ChIP-seq
[x]	[ ]	Flow cytometry
[x]	[ ]	MRI-based neuroimaging
